# Supplementary material for: Structural insights into MIC2 recognition by MIC2-associated protein in Toxoplasma gondii
Source: Commun Biol. 2023 Aug 31;6:895. doi: 10.1038/s42003-023-05277-0 (PMC10471735; doi:10.1038/s42003-023-05277-0)
Supplement: Supplementary file 2 — Supplementary Figures [file 42003_2023_5277_MOESM2_ESM.pdf]

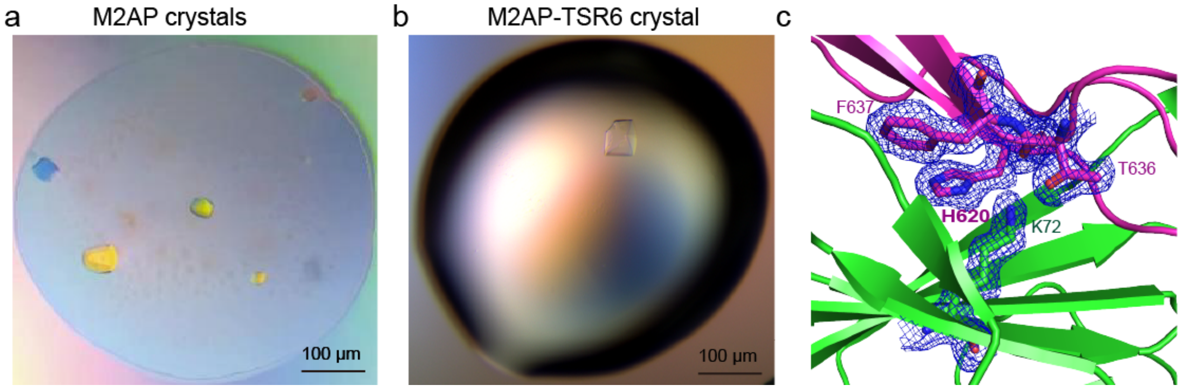

Supplementary Figure 1. The crystals and electron density map.

a-b. M2AP and M2AP-TSR6 crystals. c. The 2mFo-DFc electron density map of the key residues in the complex. The residues His-620, Thr-636 and Phe-637 in TSR6 and K72 in M2AP are shown as sticks. 2mFo-DFc contoured at 1σ (blue).

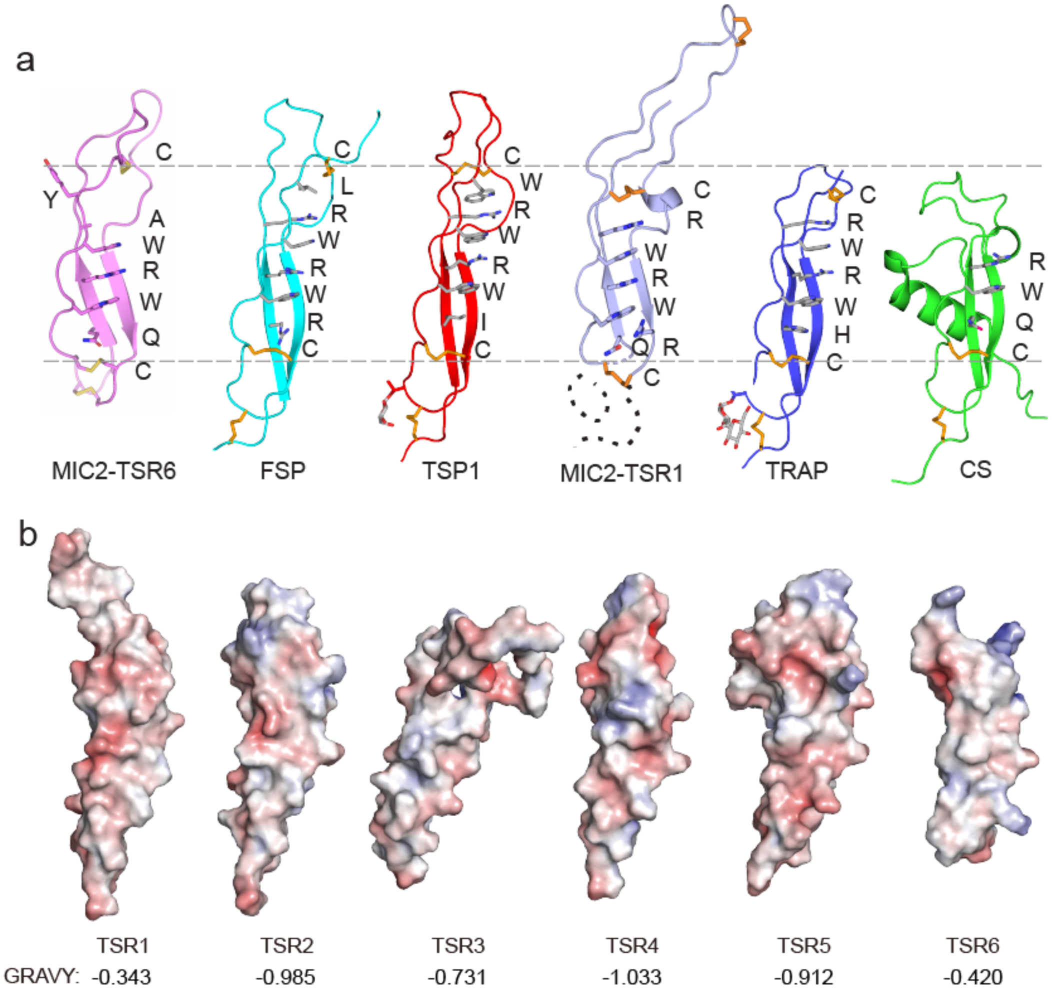

Supplementary Figure 2. The TSR domains.

a. Comparison of MIC2 TSR6 and other structurally determined TSRs. Layered residues for each TSR domain are shown and labelled. Dashed lines mark the upper and bottom disulfide bonds of the layer. b. Electrostatics of TSR6 and other TSR domains within MIC2. The M2AP-binding face is shown for TSR6 and other TSR domains are shown in identical orientations. TSR1-5 structures are from the full-length of AlphaFold MIC2 model. Electrostatic solvent accessible surfaces were calculated with scale of [−5 (red) to +5 (blue) kT/e].

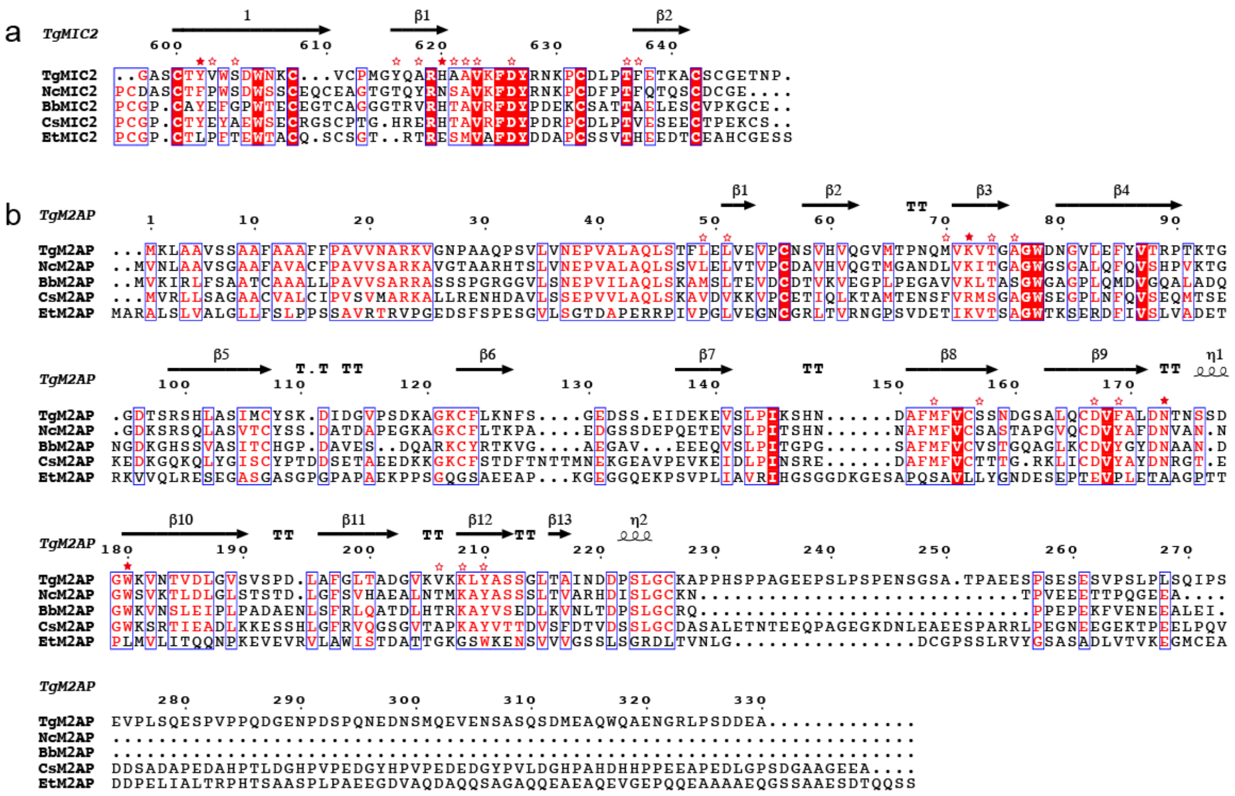

Supplementary Figure 3. Sequence alignment of TSR6 (a) and M2AP (b).

Secondary structures of tgTSR6 and tgM2AP are highlighted on the top of the alignment, respectively. Residues involved in direct hydrogen bond interactions are shown as solid star (above the alignment), while other residues within the interface are shown as empty star. *Tg, Nc, Bb, Cs, Et* stand for *Toxoplasma gondii, Neospora caninum, Besnoitia besnoiti, Cystoisospora suis, Eimeria tenella*, respectively. The EtMIC1 and EtMIC2 were named as EtMIC2 and EtM2AP for clarity of alignment. Red background means highly conserved position, while red letter means partially conserved position.

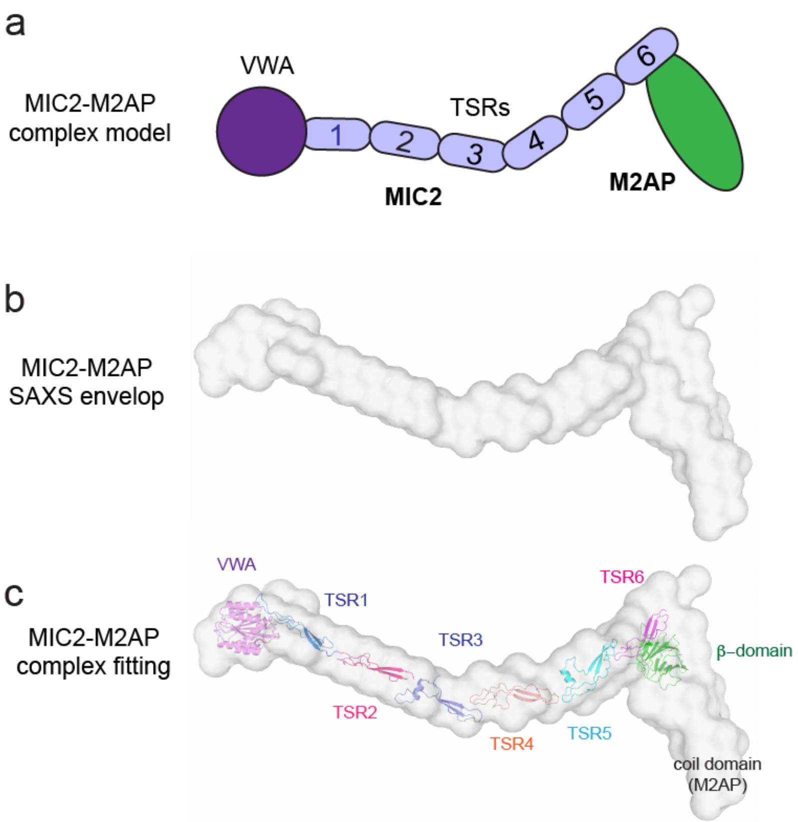

Supplementary Figure 4. Full-length MIC2-M2AP envelope and fitting model.

a and b. The complex model and SAXS envelop showing extended conformation for MIC2 (Ref. 9). c. Fitting result of previous VWA-TSR1 (ref. 9), TSR6-M2AP (this study) crystal structures, and TSR2-5 models (from AlphaFold) into the SAXS envelop model.

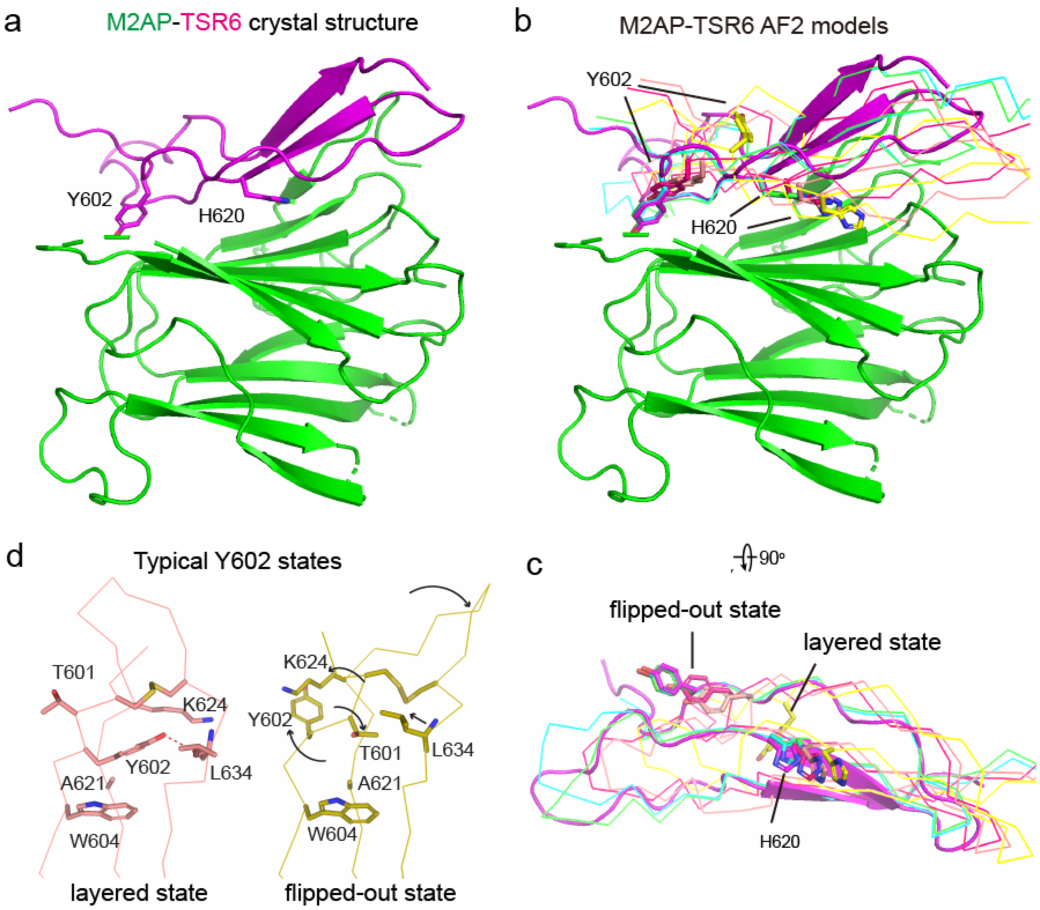

Supplementary Figure 5. The predicted AlphaFold complex models and dynamics of Y602.

a. The determined crystal structure of MIC2-M2AP complex in current study. Two key residues are shown with sticks. b-c. The predicted complex models are superimposed with the crystal structure on M2AP. TSR6s in models are shown as ribbon and the M2APs are hidden for clarity. In panel c all M2APs are hidden to view the positions of Tyr-602 and His-620. d. The dynamics of Tyr-602 between layered state and flipped-out state. Tyr-602 and nearby residues involving in conformational changes are shown as sticks, the arrows indicate movement or rotation direction.
