# Supplementary material for: Structural insights into MIC2 recognition by MIC2-associated protein in Toxoplasma gondii
Source: Commun Biol. 2023 Aug 31;6:895. doi: 10.1038/s42003-023-05277-0 (PMC10471735; doi:10.1038/s42003-023-05277-0)
Supplement: Supplementary file 3 — Description of Additional Supplementary Files [file 42003_2023_5277_MOESM3_ESM.pdf]

Description of Additional Supplementary Files

File name: Supplementary Data 1
Description: Source data for figure 3

File name: Supplementary Data 2
Description: Source data for figure 4b
